# Transactions of the Association of Fellows and Licentiates of the King's and Queen's College of Physicians in Ireland

**Published:** 1818-04

**Authors:** 


*Transactions of the Association of Fellows and Licentiates of the King's and Queen's College of Physicians in Ireland.* One Vol. Octavo, pp. 504, with Plates.

We have long been astonished, that from the metropolis of the Sister Kingdom some Medical Journal or Transactions did not emanate; knowing as we do, that Medical Science is there cultivated with a zeal and a talent, not exceeded in London or Edinburgh. We, therefore, hail the present work as an omen of the light which Ireland will shed on the dark paths of pathology; and if she proceeds as she has begun, we can pretty confidently predict,

---

that her "*Transactions*" need not shrink from a comparison with those of England and Scotland in the brightest days of their medical glory.

"The science and the practice of physic have owed so much of their advancement, during the last half century, to *Public Journals*, and the Transactions of various learned Societies, that it were now superfluous to conciliate the indulgence of the profession towards a new work of this miscellaneous kind. The spirit of research is obviously promoted by whatever tends to open fresh avenues, or multiply opportunities of communication among the inquisitive and well-informed; detached fragments of knowledge are thus brought to light, which might otherwise have been lost or overlaid by extraneous matter; and a common sympathy and co-operation obtained in the labours of professional improvement." *Preface.*

With this preliminary quotation, in the sentiments of which we fully agree, we shall proceed to our analytical labours, in our usual style of brevity and concentration.

Art. 1. The first case, by Dr. John Crampton, is an ulceration and rupture of the stomach, attended with some severe and uncommon symptoms. A young lady of sallow complexion, and "regular as to female health," but who had been subject occasionally to "a diseased state of the stomach," was seized at three o'clock, P. M. with a sudden spasm, succeeded by agonizing pains all over the abdomen, but without nausea. Notwithstanding that a variety of remedies were tried; for instance, general and local bleeding, fomentations, purgatives, glysters, opiates, &c. she experienced no relief, and expired in twelve hours.

*Dissection.* Stomach flaccid and empty, seemingly divided into two cavities "by a band of contraction" across its anterior surface. This contraction was occasioned by schirrous indurations which followed the same direction. The contents of the stomach had escaped through a round aperture, situated at the union of the cardiac and pyloric portions. The perforation was circular, and about the size of a large pea, apparently the result of an ulcer on the mucous surface. The ulcer was hollow, nearly the size of a shilling, and "had the appearance as if it had been made with caustic." Marks of recent inflammation throughout the whole peritoneum. Dr. Crampton does not seem to have entertained the least idea of any poisonous substance having been swallowed. Yet the above phænomena are so unlike what take place in spontaneous ulcerations of the stomach, that we confess the case excited in our minds some suspicions that an acrid substance had produced the fatal lesion.

ART. 2. The second paper is on *Abdominal Tumours*, by DR. STOKER. The cases, though interesting, are of a nature not to be easily, or perhaps usefully, analysed. Dr. Stoker, however, appears to be a gentleman of solid understanding and keen observation.

ART. 3. *Suppuration of the Liver*, by DR. J. O'BRIEN. About eight days before admission into hospital, Bridget Lawler, aged 18, had made a hearty dinner on salt pork and greens, followed by copious draughts of butter-milk; after which she felt a hard lump in her stomach which did not leave her. When seen by Dr. O'Brien, she had enormous swelling and tension of the whole epigastric region, with pain in the right side, much aggravated by inspiration or pressure. The tumour presented no resistance to the touch; countenance pale and anxious; pulse small and feeble, about 120. By strong purgatives, fomentations, blisters, &c. the pain was much relieved, and by the third day the abdominal tension was so far reduced, that the limits of the abscess could be ascertained. On the seventh day, her mouth was a little sore; the epigastric tumour stationary. An incision was made, and four pints of dark brown or coffee coloured fluid were discharged. On the sixth day after the operation, the patient died. On dissection, the intestines were found perfectly sound; the stomach adhered, about the middle of its lesser arch, to the base of the abscess, which involved two-thirds of the substance of the liver. The parietes of the abscess internally presented a jagged appearance, as is usual in all extensive suppurations of that organ. In Dr. O'Brien's observations on this case, we do not see any thing very interesting. We cannot agree with him, that opening an hepatic abscess when it points outwardly is of little benefit. We have seen many recover from the operation who could not possibly have otherwise survived. It has been denied, that the mouth can be made sore with mercury after suppuration has taken place in the liver. We know to the contrary, and the foregoing case affords a proof.

ART. 4. *Dissections of habitual Drunkards.* By DR. S. BLACK. The *first* was a young man, 27 years of age, who had drunk hard for two or three years. He became affected with gastric complaints, and finally with dropsy. He had been twice tapped before death, and his feet and legs were anasarcous.

*Dissection.* Liver not more than one-third its natural size; substance felt hard and rigid between the fingers; and was throughout full of small, hard tubercles; gall-bladder shrivelled, and containing scarcely any bile; pylorus rather thick and indurated.

*Second Case.* A shoe-maker, 63 years of age, had been an habitual drunkard for fifteen years; during which time he enjoyed tolerably good health. For the last eighteen months affected with a variety of gastric complaints, with obstinate costiveness, pyrosis, and vomiting. For the last six months, scarcely any solid food could be taken. A hard ridge could be felt stretching across from the right to the left hypochondrium;* legs and feet œdematous.

*Dissection.* Considerable watery effusion in the cavity of the abdomen. *Liver* considerably enlarged, with tubercles interspersed on the surface, and in the substance; gall-bladder pale and empty; spleen diminished and rigid. *Stomach* so contracted that it would not have contained a turkey egg; its coats indurated and thickened to an extraordinary degree, resembling cartilage. The interior surface abounded with several fungous excrescences, from which a brownish fluid oozed out.

From these Dissections we only learn that the structure as well as the functions of the liver may be oppositely, but still morbidly affected by the same noxious ingurgitation.

Art. 5.—Dr. Black, of Newry, relates a remarkable Case of a Medullary Tumour, which appeared to spring originally from the mesentery; but before the death of the patient, was enlarged to the size of a man's head, extending from the left hypochondrium to the os ilium. Dropsical effusion in the abdominal cavity preceded the fatal event. No medicine, of course, had any salutary effect.

Art. 6.—*A Case, shewing the Influence of Hepatic Disease over the Functions of the Uterus.* By Thomas Mills, M. D.

Bilious vomiting, head-aches, heat or pain in both the hypochondria, constipation of the bowels, and other symptoms, indicative of morbid actions in the liver and

* Our Irish brethren are fond of the term "Hypochondre." We think the Latin full as elegant as the French.

of imperfect digestion, first presented themselves; these were succeeded by amenorrhœa, a purulent discharge from the bladder and vagina, and finally, by marks of a broken constitution.

From the order and succession of the symptoms, it appears, that, at the onset of this attack, there was a vitiated secretion of bile, arising from disordered actions in the liver; imperfect digestion was the consequence, which, by its long continuance, so impaired the general health, as to cause amenorrhœa. The vitiated state of habit thus produced, not unfrequently excites in organs, predisposed to disease, new and irregular actions, especially where the constitution is scrofulous; and hence, in the present instance, the purulent discharge from the bladder and vagina. Leeches and blisters to the right hypochondrium and epigastrium, abated and corrected the increased and irregular action of the liver; while daily and repeated cathartics removed large quantities of fæcal and bilious matter from the alimentary canal. With the restoration of the powers of digestion, health returned and also the menstrual discharges.

Art. 7. A very long Case of Hydrocephalus Internus in an adult, is here related by Dr. William Brooke. It was treated principally by opium, and subsequently by mercurials in conjunction. It proved fatal; and we believe the practice pursued will not now be imitated by any medical man on this side of the water. As the case occurred seventeen years ago, the pathology was not so well understood, and the plan of early evacuations not then in vogue.

Art. 8.—*On the Use of Oxigen Gas in Angina Pectoris.* By Robert Reid, M. D.

We consider this case to be one of *Asthma Arthriticum*, an exquisite instance of which we have now under our care, the symptoms of which correspond with those related by Dr. Reid. The patient was a gentleman, ætat. 66, of low stature, corpulent habit, and short neck. He had lived freely. The following is a specimen of his paroxysms.

"He said he slept remarkably easy during the night; but that on waking he perceived something like thick phlegm obstructing his breathing, and which he was not able to expectorate. This difficulty gradually increased, attended with a rattling noise, until he was bled, which instantly relieved him. When the paroxysm was at its height, his extremities became cold; his pulse not perceptible at the wrist, and these symptoms were soon followed by profuse cold perspiration all over the surface of his body. He now complained of frequent catchings in his breathing like deep sighs, and expectorated much of a dark coloured matter."

These symptoms were often accompanied by intermissions and other irregularities in the pulse, great anxiety of countenance, with a bluish tinge thereon. All these phænomena are present in our own patient, and he is always relieved by immense doses of laudanum, æther, camphor, and ammonia; sometimes we are forced to bleed, and often to blister. To these we add very hot pediluvia, and the inhalation of warm vapour of hot water. These give relief.

In Dr. Reid's case, the inhalation of oxygen gas seemed to moderate the paroxyms, but the patient expired in one of them, which, as usual, attacked him on awaking from sleep. Our patient is so much afraid of a sound sleep, that he rarely ventures to lie down in bed, but sits nearly upright all night; and lies down awhile after the period of attack is gone by.

### Art. 9.—*On the Nature and Treatment of Tetanus.* By Robert Reid, M. D.

Considering the length of time which Dr. Sanders, of Edinburgh, has spent in teaching and demonstrating the affections of the medullar oblongata and spinal cord in spasmodic diseases, we cannot regard this paper of Dr. Reid's in any other light, than as a corroboration of the ingenious and truly original investigations of the Edinburgh Lecturer.

It has long qeen observed, that in tetanus the natural functions are little affected; and the same may be said of the intellectual functions, and those muscles and organs supplied by the nerves of sense. These considerations naturally lead to the conclusion, that the thoracic and abdominal viscera are not primarily affected, and that the origin of the disease is not in the nervous substance supplying those organs. In short, that the cerebral and ganglionic systems are only drawn in *subsequently*, and that the spinal cord is the original and principal seat of tetanus.

*Case in elucidation.* A boy, ætat. 14, after receiving a severe burn in the toes of the right foot, was exposed to the vicissitudes of the weather in the month of February. He was seized four or five days afterwards with tetanus, and died in thirty-six hours.

*Dissection.* Viscera of the abdomen and thorax perfectly sound, as were all the muscular parts. On opening the spine, *from the back part,* and on raising the nervous mass (with its dura mater entire) from the spine,

"There appeared a considerable effusion of blood into the cellular tissue, connecting it to the upper lumbar and lower dorsal vertebræ. A similar effusion occurred also along the bodies of the upper dorsal and two inferior cervical vertebræ. On slitting up the dura mater on the *anterior* surface, the nervous mass appeared highly vascular, and the vessels of every description remarkably tortuous. The only appearance in the nervous substance itself, was a deeper tinge than natural in its cortical and medullary parts."

From these appearances corresponding with the investigations of Dr. Sanders, it follows that tetanus is radically an inflammatory disease. But general blood-letting here will not be near so efficacious as local abstractions of blood from the spine—blisters—purgatives—and finally, mercury and opium to equalize the balance of the circulation.

Art. 10. *On the good Effects of Oil of Turpentine in three Cases of Melena.* By William Brooke, M. D.

This is a diffusely written paper, and the cases detailed with rather too much minuteness. In the first case, there was evidently derangement of the hepatic system; for the tar-like and inodorous stools were twice changed for yellow and natural motions, as soon as the gums were made sore by mercury.

"His strength and appetite, however, improved, and immediately on the salivation coming on, the morbid appearance of the stools entirely ceased; the mouth continued sore for about two weeks, during which time, the stools were natural, and often extremely yellow." 127.

He caught cold after this; the melena returned, and produced alarming symptoms. Twenty drops of oil of turpentine were given in draught thrice a day, which, in two days, removed the disease. The gentleman's constitution, however, was gone; he had no digestive powers left; every thing turned into acid and flatulence; œdema of the lower extremities took place, and he died.

In the second case, the discharges were most decidedly bilious; for "the stools though black like tar, tinged the vessel of a deep olive, and on dilution, became yellow." Twenty-five drops of the oil were ordered thrice a day, which soon removed the disease, and changed the alvine evacuations.

Dr. B. enters into a long discussion on the question whether melena be a biliary or sanguineous discharge; and concludes that it is sometimes the one, and sometimes the other. We are inclined to think, that it is much more frequently a *morbid* secretion from the liver or *intestines,* than an effusion of blood. Hence it may still be a secretion, and yet incapable of communicating a yellow tinge to an aqueous menstruum. The remedy in question deserves a further trial, and promises to be useful.

ART. 11. *On Dropsy and Apoplexy.* By WILLIAM STOKER, M. D.

THIS is a very interesting paper, though the case which forms its basis is prolixly detailed.

Mr. T. W. a proprietor of lead works, and exposed to rapid alternations of heat and cold, had been affected, for eight months, with an eruption, [lepra vulgaris] which extended over the right leg, and during the same time had suffered from nausea, cardialgia, and vomiting. *He bathed the affected leg daily in cold sea water.* Present symptoms. 21st of February, 1817. Quick laborious respiration; sense of suffocation; hard cough; tightness across the chest; with anasarca over the face, trunk, and extremities. *The leprous eruption quite dry.* Pulse quick; urine scanty. Bleeding, purging, and a blister to the sternum gave some relief. The blood was sizey, and the symptoms returning, rendered venæsection again and again necessary. The affection of the respiration having a little subsided, great palpitation of the heart came on, which was removed by a blister, and some antispasmodics. He got nearly well; but on the 31st of March following, a new train of symptoms were developed. His arms became spasmed; head drawn back, limbs rigidly convulsed; countenance distorted and livid; foaming at the mouth; jaws locked; eyes protruded; vessels of the head and neck distended; pulse strong and full; breathing stertorous. The temporal arteries on both sides were obliged to be opened. Copious detractions of blood; purgatives and mercurials were employed, and he recovered.

Dr. Stoker enters into a long dissertation to shew, that the principal office of the liver is "connected with the secretion of fat." But those who have attentively studied the conversions and mutations of diseases, as ably elucidated by the writings of Ferriar and Parry in particular, can have little difficulty in tracing the foregoing trains of symptoms to the incautious application of cold water to the eruption on the legs. The various determinations to different organs that succeeded are easily explicable on this principle.

ART. 11. Dr. RICHARD GRATTAN relates in this paper, a case of very deplorable chronic rheumatism cured by bandages, on the plan of Dr. Balfour. Mrs. G. 35 years of age, was exposed to cold after her accouchement, and in a few weeks experienced a loss of power in her feet and legs, attended with numbness. These symptoms continued, and at length arrived at the following acmé, in spite of various remedies. The feet were both distorted, being permanently drawn inwards and downwards, so as to hide completely the inner ankle, while the outer one was protruded considerably. The joints of the great toes were swelled and inflamed, the skin appearing smooth, polished, and of a bright red colour. The slightest touch produced the most exquisite pain, and the pressure even of the bed-clothes was intolerable. The pains were so acute as to deprive her of rest; but her appetite was good, and her general health unimpaired. The feet were rolled in soft wool, and then the bandages were applied, as recommended by Dr. Balfour. They were only kept on during the day; and in the evening, the feet were immersed in warm salt and water. "In the course of about a week, a rapid and decided improvement was observed; the pains were greatly relieved; the swellings of the feet had diminished; the feet had become more straight; and their skin had resumed its natural colour." In short, by a perseverance in these measures alone, she recovered.

We certainly have ourselves seen good effects from tight bandaging in chronic rheumatism; but have also been much disappointed in its use. It may be tried with advantage in conjunction with other means, or after they have failed.

ART. 12. *Hydrocephalic Fever.* By Dr. CRAMPTON. THIS is a very interesting and able paper. Dr. Crampton animadverts on the impropriety of the term hydrocephalus, almost in the words of our "Observer," No. xvi. p. 273, as will be seen by the following passage.

"The name hydrocephalus, indiscriminately attributed to acute diseases, affecting the cerebral organs, would seem to imply, that in every instance an effusion of fluid had actually taken place; such a nosological title appears to me, as likely to lead, in many instances, to erroneous pathological views, as well as to considerable sources of mistake in the treatment. Practitioners acting on this impression, are apt to confine their efforts chiefly, if not altogether, to such remedies as promote the absorption of fluid. But as dropsy in other cavities, according to the ingenious observations of Dr. Blackall,* is often preceded by inflammatory symptoms; it is not improbable, that an inflammatory state of the membranes lining the cerebral cavities, or of the exhalents which open into the ventricles, may precede the effusion of fluid; and if this antecedent state can be observed by the practitioner sufficiently early, and treated as other acute inflammatory diseases, success may probably attend his efforts."

The youth, whose case is here related, had all the premonitory symptoms of hydrencephalus; that is, the symptoms of increased excitement in the vessels or coverings of the brain and spinal cord, and they were most vigorously, ably, and successfully opposed by leeching, arteriotomy of the temples; cold lotions to the head; general blood-letting and mercury, both to purge and salivate. With the mercury was generally combined opium, as recommended by Dr. Cheyne in his late valuable publication. The disease was obstinate and violent; the issue long doubtful; but the success at length complete. This youth, 12 years of age, took between the 15th of September and the 23d of October, four hundred and two grains of calomel. He was in a state of ptyalism for a considerable time before the disease was entirely subdued.

"Opium, says this judicious physician, in mitigating the sufferings of the patient, and in diminishing the morbid animal sensibility, promises to do much in saving the cerebral organs from the general destruction which awaits them; nor will this opiate treatment be found to interfere with the action of purgatives in keeping up a properly regulated condition of the bowels." 190.

A paper on the epidemic fevers of Dublin, by Dr. E. Percival, occupies nearly half the volume, and is so important, that we shall make it the subject of a separate analysis. Here then we pass it over, and conclude our present Review.

We cannot close this paper without expressing our conviction that the present volume may fairly competite with any volume of a similar kind published in this country within our memory.

---

* Blackall on Dropsies.

